# A functional inflammasome activation assay discriminates between genetically proven caps patients and patients with low penetrance NLRP3 variants

**DOI:** 10.1186/1546-0096-12-S1-P74

**Published:** 2014-09-17

**Authors:** Nikolaus Rieber, Alina Gavrilov, Theresa Endres, Dominik Hartl, Jasmin Kümmerle-Deschner

**Affiliations:** 1 Children's hospital, Tübingen, Germany

## Introduction

The cryopyrin-associated periodic syndromes (CAPS) are characterized by recurrent episodes of systemic inflammation. CAPS is caused by mutations in the *NLRP3* gene encoding cryopyrin, an important component of the NLRP3 inflammasome that activates caspase-1 resulting in inflammation by excessive production of IL-1β and others. A diagnostic dilemma is often encountered in patients with unspecific inflammatory symptoms like fatigue, muscle pain, arthralgia or slight hearing loss and low penetrance variants in *NLRP3 / CIAS* with an inconsistent clinical phenotype. The analysis of IL-1β in the serum did not prove to be a valid diagnostic test in these individuals.

## Objectives

In this study we sought to investigate, if a functional inflammasome activation assay discriminates between genetically proven CAPS patients, patients with low penetrance *NLRP3* variants and healthy controls.

## Methods

The study population consisted of 16 patients with genetically proven Muckle-Wells syndrome, 9 patients with low penetrance *NLRP3* variants (V198M, Q703K and E627G) and 14 healthy controls. Concentrations of IL-1β, IL-1α, IL-18, and Caspase-1 were quantified in cell culture supernatants after inflammasome stimulation with LPS and LPS + ATP for several timepoints.

## Results

After 4h of LPS stimulation, secretion of NLRP3 inflammasome products (IL-1β, IL-1α, IL-18) and Caspase-1 were potently increased in MWS patients, whereas there was no increase in low penetrance *NLRP3* variants and healthy controls (for IL-1β p < 0.001 and p < 0.001, respectively). Minor differences were still detected at later timepoints and for LPS + ATP stimulation.

## Conclusion

Our functional inflammasome activation assay discriminates between genetically proven CAPS patients and patients with low penetrance *NLRP3* variants. This assay might add to the decision, which individuals presumably benefit from an anti-IL-1 therapy.

## Disclosure of interest

N. Rieber Grant / Research Support from: Novartis Research Grant, A. Gavrilov: None declared., T. Endres: None declared., D. Hartl: None declared., J. Kümmerle-Deschner Grant / Research Support from: Novartis Research Grant.

